# From Management to Cure: The Shifting Paradigm in HIV and Chronic Viral Hepatitis

**DOI:** 10.3390/ph18071034

**Published:** 2025-07-11

**Authors:** Daniel Sepúlveda-Crespo, Salvador Resino

**Affiliations:** Centro Nacional de Microbiología, Instituto de Salud Carlos III, Carretera Majadahonda-Pozuelo, Km 2.2, 28220 Madrid, Spain

The management of human immunodeficiency virus (HIV) and chronic viral hepatitis (HBV, HCV, and HDV) infections continues to pose a significant global health challenge [1]. These infections frequently co-occur due to shared transmission routes. Collectively, they are a leading cause of morbidity and mortality worldwide, contributing to cirrhosis, end-stage liver disease, and hepatocellular carcinoma in coinfected patients [2,3]. Nevertheless, substantial progress in their treatment has been achieved in recent years. A better understanding of the pathophysiology of these diseases, coupled with the development of novel therapies, has created new therapeutic opportunities for both patients and healthcare providers [4,5,6].

HIV treatment has evolved notably since the introduction of the first antiretroviral drugs [7]. Combination antiretroviral therapy (ART) has transformed HIV from a fatal disease into a manageable chronic condition, allowing people with HIV to achieve a near-normal life expectancy [8]. This progress stems from the development of diverse drug classes that target the virus in various stages of its life cycle [9]. Similarly, hepatitis C treatment has been revolutionized by the advent of direct-acting antivirals (DAAs), which achieve cure rates exceeding 95% and have made the elimination of the virus a feasible goal [10,11]. Despite this therapeutic success, a major challenge remains in identifying the large population of asymptomatic, undiagnosed individuals and ensuring their access to treatment [10,11]. For HIV, a cure remains elusive due to the persistence of latent viral reservoirs [12]. In the case of hepatitis B, current treatments effectively suppress viral replication but rarely achieve a cure, as a stable viral form—covalently closed circular DNA (cccDNA)—persists in the liver [5,13].

Despite these advances, challenges remain. Drug resistance, long-term toxicity, and the management of coinfections continue to be areas of active research [14]. The cumulative impact of both HIV infection and long-term ART on end-organ health represents a concern. In this Special Issue, the systematic review and meta-analysis by Strauss et al. [15] provides compelling evidence of this ‘double risk’, showing that both HIV infection and ART can contribute to hepatic dysfunction. Cicalini et al. [16] investigate the optimization of ART in a real-world clinical setting. Their findings indicate that switching to a combination of doravirine/lamivudine/tenofovir disoproxil fumarate (DOR/3TC/TDF) is a safe and effective strategy, maintaining viral suppression, improving CD4+ T-cell counts, and demonstrating a favorable metabolic profile. Another critical focus addressed in this Special Issue is the management of coinfection. Marin et al. [17] report data from a Romanian cohort showing that, while HIV treatment remains highly effective in patients with HBV or HCV coinfection, these individuals exhibit poorer hepatic and pancreatic outcomes. These results highlight the need for integrated care and close monitoring in this vulnerable patient population.

Beyond the optimization of existing drugs, the path to a cure requires novel strategies. For HIV, one promising approach involves targeting the virus at the transcriptional level. In this Special Issue, Khatkar et al. [18] investigate small molecules that inhibit HIV transcription by disrupting the Tat-transactivation response element (TAR) interaction. They propose a “block and lock” strategy aimed at silencing the provirus. This transition from conventional drug discovery to cutting-edge technology is comprehensively reviewed by Sun and Wang [19]. Their work traces the trajectory of HIV drug development, from its early stages to the emergence of advanced technologies, and highlights the potential of gene-editing tools like CRISPR-Cas9 for achieving viral eradication. For chronic hepatitis B, where a functional cure remains elusive, understanding the host immune response is essential. Costa et al. [20] review the role of immune checkpoint receptors (such as PD-1, CTLA-4, and Tim-3) in chronic hepatitis B, providing a rationale for the development of immunotherapies aimed at restoring antiviral immunity and improving clinical outcomes.

Finally, the control of these epidemics also relies on the availability of rapid and accessible diagnostic tools. Addressing this need, Vidal-Alcántara et al. [21] describe a lateral flow assay for detecting the HCV core antigen. This type of diagnosis could greatly facilitate the diagnosis of HCV at the point of care, particularly in resource-limited settings, thereby improving case detection and linkage to treatment.

In conclusion, the recent advances in the treatment of HIV and viral hepatitis represent a remarkable scientific achievement, offering substantial hope for patients. Novel therapies, a deeper understanding of viral immunopathogenesis, and advances in diagnostic tools are moving us closer to the goal of eliminating these infections as public health threats. This Special Issue highlights the continued innovation in the field. The concerted efforts of researchers, clinicians, and the pharmaceutical industry will continue to drive progress toward a future where these devastating diseases can be effectively managed, controlled, or ultimately cured.
